# Long-term outcomes after penile prosthesis placement for the Management of Erectile Dysfunction: a single-Centre experience

**DOI:** 10.1186/s12610-021-00123-x

**Published:** 2021-03-04

**Authors:** Valentine Frydman, Ugo Pinar, Maher Abdessater, William Akakpo, Pietro Grande, Marie Audouin, Pierre Mozer, Emmanuel Chartier-Kastler, Thomas Seisen, Morgan Roupret

**Affiliations:** 1 Department of Urology, Sorbonne Université, GRC n 5, Predictive Onco-Urology, APHP, Hôpital Pitié-Salpêtrière, F-75013 Paris, France; 2 Department of Urology, Sorbonne Université, APHP, Hôpitaux universitaires Pitié-Salpêtrière-Charles Foix, F-75013 Paris, France; 3 Department of Urology, Sorbonne Université, APHP, Hôpital Tenon, F-75013 Paris, France

**Keywords:** Erectile dysfunction, Outcomes, Intraoperative events, Penile prosthesis, Functional results, dysfonction érectile, événements peripératoires, prothèse pénienne, résultats fonctionnels

## Abstract

**Background:**

Penile prothesis (PP) is the gold-standard treatment of drug-refractory erectile dysfunction (ED). While postoperative outcomes have been widely described in the literature, there are few data about patient satisfaction and intraoperative events. We aimed to assess long-term patient satisfaction and perioperative outcomes after PP implantation in a single-centre cohort of unselected patients using validated scales.

**Results:**

A total of 130 patients received a PP (median age: 62.5 years [IQR: 58–69]; median International Index of Erectile Function (IEEF-5) score: 6 [IQR: 5–7]). Median follow-up was 6.3 years [IQR: 4–9.4]. Thirty-two (24.6%) patients underwent surgical revision, of which 20 were PP removals (15.4%). Global PP survival rate was 84.6% and previous PP placement was a risk factor for PP removal (*p* = 0.02). There were six (4.6%) non-life-threatening intraoperative events including two which resulted in non-placement of a PP (1.5%). EAUiaic grade was 0 for 124 procedures (95.4%), 1 for four procedures (3.1%) and 2 for two procedures (1.5%). Of patients who still had their PP at the end of the study, 91 (80.5%) expressed satisfaction.

**Conclusions:**

PP implantation is a last-resort treatment for ED with a satisfactory outcome. PPs are well accepted by patients.

## Résumé

**Contexte:**

La prothèse pénienne (PP) est. le traitement de référence de la dysfonction érectile (DE) réfractaire aux médicaments. Le but de ce travail est. d’évaluer les résultats à long terme chez les patients traités par prothèses péniennes (PP) dans notre hôpital.

**Résultats:**

Un total de 130 patients a eu une pose de PP (âge médian: 62,5 ans [IQR: 58–69]; score médian de l’indice international de la fonction érectile (IEEF-5): 6 [IQR: 5–7]). Le suivi médian était de 6.3 ans [IQR: 4–9.4]. Trente-deux (24,6%) patients ont eu une reprise chirurgicale, dont 20 étaient des retraits de PP (15,4%). La durée de vie global des PP était de 84,6% et la pose antérieure de PP était un facteur de risque pour l’ablation (*p* = 0,02). Il y a eu six (4,6%) événements peropératoires ne mettant pas la vie en danger, dont deux qui ont empêché la pose de PP (1,5%). Le score EAUiaic était de 0 pour 124 procédures (95,4%), 1 pour quatre procédures (3,1%) et 2 pour deux procédures (1,5%). Parmi les patients qui avaient encore leur PP à la fin de l’étude, 91 (80,5%) ont exprimé leur satisfaction.

**Conclusions:**

L’implantation de PP est. un traitement de dernier recours pour la dysfonction érectile avec un résultat satisfaisant. Les PP sont bien acceptés par les patients.

## Introduction

Erectile dysfunction (ED) is a frequent condition in men which increases with age. More than 150 million men currently complain of ED and this number is expected to rise in the near future [1]. Penile prosthesis (PP) placement is a recommended surgical treatment for drug-refractory ED regardless of aetiology [2].

In France, although PP implantations doubled between 2006 and 2013, less than 10% of surgeons perform a high volume of PP placements (i.e. > 20 cases/year) [3]. PP placement costs are covered by the health insurance and therefore every patient has access to this procedure regardless of their condition or surgical motive.

Postoperative outcomes and patient/partner satisfaction with PPs have already been described, with positive results in selected patients [4–6]. However, there are no data regarding intraoperative events, especially since the EAU Intraoperative Adverse Incident Classification (EAUiaic) was validated [7]. Moreover, only a few studies have assessed patient satisfaction using validated scales such as the Erectile Dysfunction Inventory of Treatment Satisfaction (EDITS) scale, many years ago [8, 9].

Our aim was to assess long-term patient satisfaction, but also perioperative outcomes after PP implantation in a single-centre cohort of unselected patients.

## Material and methods

### Patients and data collection

All men who underwent surgery for inflatable PP placement between 2004 and 2019 were included. A PP was proposed in cases of drug-refractory ED as assessed by the International Index of Erectile Function (IIEF-5) score. There were no exclusion criteria. Patient satisfaction was assessed annually and prospectively using the EDITS patient version score [10]. Demographic, clinical and perioperative data were collected retrospectively from our clinical follow-up notes. At the time of the study, each included patient was phoned in order to improve follow-up regarding PP revision or placement, and its functionality. The same investigator retrieved the data and was not involved in the treatment of those patients.

### Surgical technique

The inflatable PP models inserted were AMS 700CX (American Medical Systems, Minnetonka, MN, USA) or Coloplast Titan (Coloplast Corp, Minneapolis, MN, USA) depending on the surgeon’s choice. In our department, only two experienced surgeons (> 10 years) performed this surgery, as reported previously [11]. Depending on the surgeon’s habits, either peno-scrotal or infra-pubic approaches were used. Perioperative risk management was handled with an intraoperative alcohol-based bath and antibiotic prophylaxis administered around 30 min before surgical incision (2nd generation cephalosporin, aminoglycoside in case of allergy). At the end of the procedure, the PP was left inflated at 80% of its maximal capacity for 72 h. If possible, patients were discharged at day 1. They were asked to avoid sexual intercourse until PP activation. They were prescribed non-opioid pain killers and daily nursing care. Six weeks after surgery, surgeon activated the PP during a face-to-face consultation. Patient follow-up consisted of an evaluation of satisfaction using the EDITS score [10] and PP function at 6, 12 months and annually thereafter.

### Outcomes

Study outcomes were patient satisfaction, PP survival rate with/without removal and perioperative complications. A patient was considered satisfied if they answered “A” or “B” to questions 1, 2, 7 and 11 on the EDITS questionnaire (Table 1). Surgical perioperative complications were assessed using the EAUiaic score [7]. Surgical postoperative complications included each surgical or medical complications after PP placement and were assessed using the Clavien-Dindo classification [12]. The quality of complications reporting was assessed using Martin’s criteria as recommended by the EAU Guidelines office panel [13]. Details of the criteria used to assess surgical outcome are shown in Table 1.

**Table 1:** EDITS questionnaire item 1,2,7 and 11

Question	Possible answers
1- Overall, how satisfied are you with this treatment?	a. Very satisfied
	b. Somewhat satisfied
	c. Neither satisfied nor dissatisfied
	d. Somewhat dissatisfied
	e. Very dissatisfied
2- During the past four weeks, to what degree has the treatment met your expectations?	a. Completely
	b. Considerably
	c. Half way
	d. A little
	e. Not at all
7- How confident has this treatment made you feel about your ability to engage in sexual activity?	a. Very confident
	b. Somewhat confident
	c. It has had no impact
	d. Somewhat less confident
	e. Very much less confident
11. Compared to before you had an erection problem how would you rate the naturalness of your erection when you used this treatment over the past four weeks in terms of hardness?	a. A lot harder than before I had an erection problem
	b. Somewhat harder than before I had an erection problem
	c. The same hardness as before I had an erection problem
	d. Somewhat less hard than before I had an erection problem
	e. A lot less hard than before I had an erection problem

### Statistical analysis

Statistical analysis was performed using R version 3.6.2. (2009–2019 RStudio, Inc.). Quantitative variables are described as median and interquartile range [IQR] and qualitative variables as number and percentage. To compare categorical variables, Pearson’s Chi^2^ test was used. The Mann-Whitney U test was performed to compare continuous variables. Survival rate was defined as the percentage of patients who did not undergo any surgery on the PP due to malfunction without removal within 36 months after initial surgery. Survival rate without removal represented patients who underwent revision with no PP removal. Global survival rate corresponded to patients who still had their initial PP at the end of the study. All tests were bilateral. Significant results were set for a *p*-value < 0.05.

### Ethics

Informed consent was signed by the patient at the first postoperative consultation. The study was approved by the local ethics committee (APHP) and was conducted according to the Declaration of Helsinki.

## Results

### Study population

A total of 130 patients were included in the study. Median age was 62.5 years [IQR: 58–69], median IIEF-5 score was 6 [IQR: 5–7] and 122 patients (93.8%) had received previous intracavernous injections. The causes of severe ED were: previous radical prostatectomy (*n* = 59, 45.4%), diabetes (*n* = 35, 26.9%) and other causes (*n* = 36, 27.7%). Overall, 76 patients received an AMS 700 CX device (58.5%) and 54 (41.5%) were implanted with a Coloplast Titan. The peno-scrotal approach was used in the majority of cases (*n* = 96, 73.8%). Of the included patients, nine (7%) had concomitant artificial urinary sphincter (AUS) placement. During the study, there was not reported death among patients. Each patient was reachable by phone (0% lost to follow-up) and median follow-up was 6.3 years [IQR: 4–9.4]. The characteristics of the patients are summarised in Table 2.

**Table 2:** Baseline characteristics of the study population

Characteristic	Study cohort (***N*** = 130)
**Age (years), median [IQR]**	62.5 [58–69]
**ASA score, median [IQR]**	**2 [1–2]**
**IIEF-5 score, median [IQR]**	6 [5–7]
**Time between ED and PP implant (months), median [IQR]**	38 [24–72]
**Primary cause of ED, n (%)**	
Diabetes	35 (26.9)
Radical prostatectomy	59 (45.4)
Priapism/penile trauma	6 (4.6)
Spinal cord injury	2 (1.5)
Peyronie’s disease	8 (6.2)
Iatrogenic	4 (3.1)
Others	16 (12.3)
**Previous ED treatment, n (%)**	
PDE5-I	69 (53.1)
Intracavernous injection	122 (93.8)
Penile prothesis	5 (3.8)
Vacuum	9 (6.9)
**PP type**, *n* (%)	
AMS 700	76 (58.5)
Coloplast Titan	54 (41.5)
**Surgical approach,** ***n*** **(%)**	
Peno-scrotal	96 (73.8)
Infra-pubic	34 (26.2)
**Follow-up (years), median [IQR]**	**6.3 ± [4–9.4]**

*ASA* American Society of Anaesthesiologists, *IIEF* International Index of Erectile Function, *PP* penile prosthesis, *ED* erectile dysfunction, *IQR* interquartile range

### Surgical outcomes

EAUiaic grade was 0 for 124 procedures (95.4%), 1 for four procedures (3.1%) and 2 for two procedures (1.5%) (Table 3). Intraoperative urethral injury resulted in cancellation of PP placement and urethral catheterisation in two patients.

**Table 3:** Surgical outcomes

	Study cohort (***N*** = 130)
**EAUiaic grade,** ***n*** **(%)**	
0	124 (95.4)
1	4 (3.1)
2	2 (1.5)
3	0 (0)
4	0 (0)
5	0 (0)
**Intra-operative complications details,** ***n*** **(%)**	
Urethral injury	2 (1.5)
Cylinder crossover	3 (2.3)
Cylinder perforation	1 (0.8)
**Postoperative complications,** ***n*** **(%)**	
Clavien 1	19 (14.6)
Clavien 2	2 (1.5)
Clavien 3	23 (17.7)
**Surgical revision,** ***n*** **(%)**	
***With PP removal***	20 (15.4)
PP infection	12 (9.2)
PP erosion	6 (4.6)
Haematoma	2 (1.5)
***Without PP removal***	12 (9.2)
Pump malfunction	5 (3.8)
Up-size requested by patient	2 (1.5)
PP malfunction	2 (1.5)
Aesthetic	2 (1.5)
Tube erosion	1 (0.8)
**36-month survival rate (%)**	80.7
**PP global survival rate (%)**	84.6
**Survival without PP removal**	37.5

*PP* penile prosthesis, *EAUiaic* EAU Intraoperative Adverse Incident Classification

Of the 130 procedures, 32 revisions (24.6%) were achieved without PP removal in 12 cases (9.2%). A major issue was pump malfunction (five revisions, 3.8%) that was corrected by pump replacement. Four revisions were performed at the patients’ request: two for aesthetic reasons (1.5%) and two for PP up-sizing (1.5%).

**Fig. 1:**
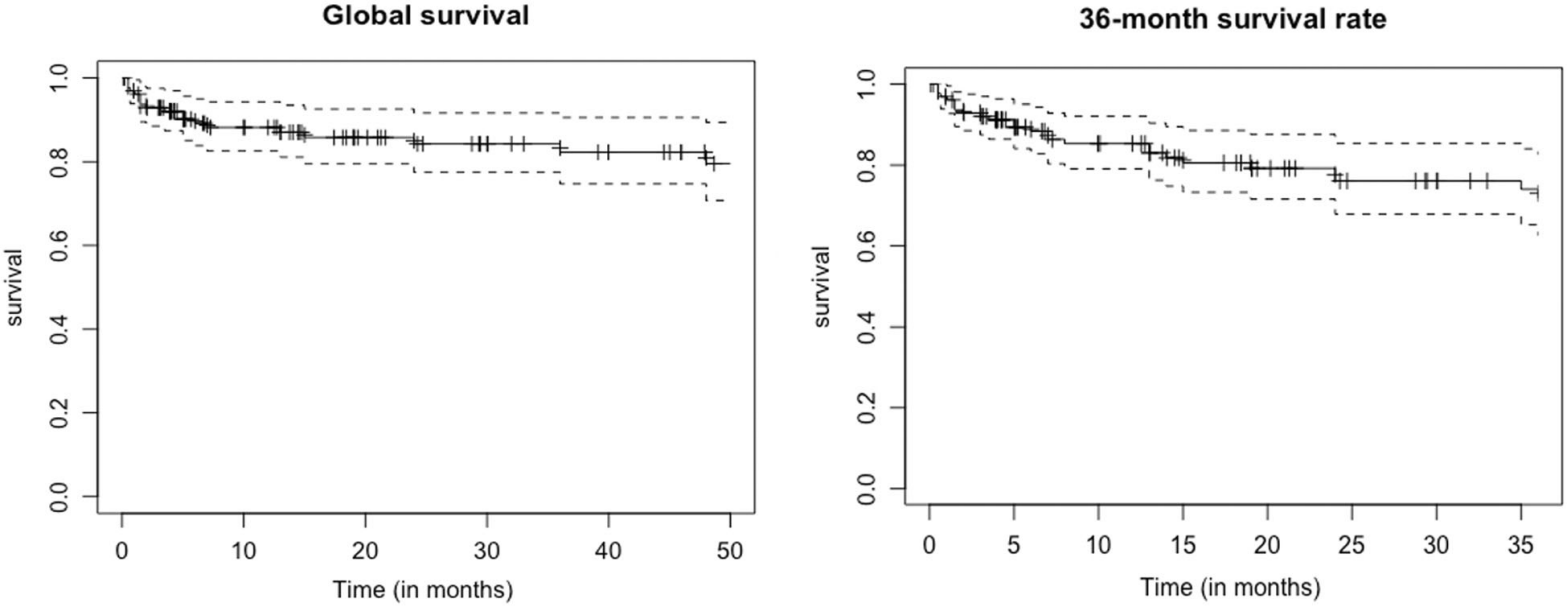
Penile implant survival rate at 3 years and global survival

The 3-year PP survival rate (*n* = 120 patients) was 80.7% (Fig. 1). A between-group analysis (surgical revision vs. no surgical revision) revealed that the peno-scrotal approach was most frequently associated with surgical revision (90.6% were peno-scrotal and 9.4% were infra-pubic; *p* = 0.02) (Table 4).

**Table 4:** Factors related to surgical revision and to PP removal

	Surgical revision (***N*** = 32)	No surgical revision (***N*** = 98)	No PP removal (***N*** = 110)	PP removal (***N*** = 20)
**Age (years), mean ± SD**^**1**^	60.2 (8.8)	62.5 (9.3)	62.2 (9.3)	60.6 (8.6)
**PP size (cm), mean ± SD**^**1**^	17.2 (1.8)	16.7 (2.4)	16.7 (2.3)	17.2 (2)
**Surgical approach,** ***n*** **(%)**^**2**^				
Peno-scrotal	29 (90.6)	67 (68.4) *	78 (70.9)	18 (90)
Infra-pubic	3 (9.4)	31 (31.6) *	32 (29.1)	2 (10)
**Type of PP,** ***n*** **(%)**^**2**^				
Coloplast Titan	15 (46.9)	39 (39.8)	45 (40.9)	9 (45)
AMS 700	17 (53.1)	59 (60.2)	65 (59.1)	11 (55)
**Previous AUS,** ***n*** **(%)**^**2**^	2 (6.3)	5 (5.1)	6 (5.5)	1 (5)
**Previous PP,** ***n*** **(%)**^**2**^	3 (9.4)	2 (2.1)	2 (1.8)	4 (20) *
**Diabetes,** ***n*** **(%)**^**2**^	15 (46.9)	35 (35.7)	39 (35.5)	11 (55)
**Radical prostatectomy,** ***n*** **(%)**^**2**^	12 (37.5)	47 (48)	53 (48.2)	6 (30)
**Hypertension,** ***n*** **(%)**^**2**^	19 (59.4)	47 (48)	54 (49.1)	12 (60)
**Smoking,** ***n*** **(%)**^**2**^	12 (37.5)	37 (37.8)	42 (38.2)	7 (35)
**Dyslipidemia,** ***n*** **(%)**^**2**^	13 (40.1)	27 (27.6)	31 (28.2)	9 (45)

*PP* penile prosthesis, *AUS* artificial urinary sphincter, *SD* standard deviation

* *p*-value< 0.05

^**1**^ **Mann-Whitney U test**

^**2**^ **Pearson’s Chi**^**2**^ **test**

Overall, 20 PPs were removed after initial surgery: 12 due to prosthesis infection (60%), six for urethral or skin erosions (30%) and two due to haematomas (10%). Global PP survival rate was 84.6% at the end of the study (Fig. 1). Of the patients who underwent PP removal (one for urethral erosion and two for haematomas), three received a second PP. Previous PP placement was the only significant factor associated with removal (*p* = 0.02) (Table 4).

Overall, there were 44 postoperative surgical complications graded according the Clavien-Dindo classification as follows: 19 were grade 1 (14.6%), two were grade 2 (1.5%) and 23 were grade 3 (17.7%).

### Satisfaction outcomes

Two months after surgery, 85 patients (66.4%) were satisfied with the results of PP placement. The two patients who did not undergo PP placement because of urethral injury were excluded from this analysis. At the end of study, 91 (71.1%) were satisfied according to the EDITS scale. The two main complaints regarding the PP were pump manipulation and the size of their penis.

## Discussion

In the current study, we evaluated surgical outcomes and patient satisfaction after PP placement at a single academic centre. Patient satisfaction was assessed prospectively from the beginning of the study strengthening our results.

We observed a high level of satisfaction, which could be slightly lower with that described in the literature [14, 15]. Our satisfaction rate may differ from literature, but it was calculated including each patient of this study regardless PP removal or revision and approached 86% for patients who still had their PP at the end of the study.

The satisfaction rate increased between the first consultation and the last follow-up; this observation seems to be correlated with a necessary period of practice for patients. Patients need to become familiar with the pump but also need to relearn how to engage in sexual intercourse. In a retrospective study, Carvalheira et al. demonstrated that sexual function was associated with male satisfaction after PP placement [16]. Factors associated with dissatisfaction included loss of penis length, retarded ejaculation, partner not satisfied and unnatural sensation because of the PP. In order to maximise functional results after PP placement, patients should be informed prior to surgery about PP manipulation, sexual intercourse modifications, altered penis sensation and modified penis length [17]. Patient selection is also a factor influencing satisfaction with worse satisfaction described in patients with Peyronie’s disease or > 70 years of age [9].

In our study, the choice of PP device was left to the surgeons and > 50% of patients received an AMS 700. Previous reports have failed to demonstrate any significant difference in satisfaction between an AMS 700 and Coloplast Titan [18]. Satisfaction rate was comparable in the two groups, while a significant level of dissatisfaction was observed with a Coloplast Titan PP concerning the appearance of the penis at erection. Our results are consistent with these findings.

Our results show that a second PP placement was a risk factor for subsequent removal. Revision surgery is associated with an increased rate of post-surgical events such as infection, chronic pain or reduced PP survival [19]. Henry et al. hypothesised that revision surgery could re-activate bacteria already present in the peri-prosthetic biofilm. These findings are in line with an increased rate of PP infection associated with a patient’s surgical history [20].

Our revision rate was superior to that reported in previous studies, [21, 22] but we did not include any specific inclusion criteria and some of our patients could be considered to be at high risk. At least 10% of our patients had already undergone previous PP placement or AUS, and almost 40% of our patients were diabetic. As a consequence, we report a 9% rate of PP infection that is consistent with previous studies including diabetic patients [23]. Ultimately, among the reasons given for revision, four were due to patient choice (aesthetic or PP size) and could explain some of our high rate of revisions.

The peno-scrotal approach was more often associated with surgical revision in our study. These results are not in line with those in the literature which have shown no difference regarding the surgical approach [24, 25]. This could be explained by the fact that all surgeries for a second PP placement were performed using the peno-scrotal approach or that second PP placements are associated with a greater risk of postsurgical events.

We used the new EAUiaic scale to describe perioperative incidents during PP placement [7]. Only two procedures were interrupted because of urethral perforation. Our overall complication rate was consistent with that of Minervini et al. who reported an overall perioperative complication rate of 4.8% [26].

Our study has several limitations. First, patients came from a single-centre and the population size was small. In addition, the median follow-up period of 1.8 years may appear low for a study assessing satisfaction, but all of our patients did attend the last consultation.

## Conclusion

PP placement for patients with treatment refractory ED was a satisfactory procedure in our population. Intraoperative outcomes were good with a EAUiaic grade 2 reported for only two patients. Moreover, long-term surgical outcomes were acceptable with an 81% PP survival at 3 years.

## Data Availability

All data generated or analyzed during this study are included in this published article.

## Abbreviations

AUS: Artificial Urinary Sphincter
EAUiaic: EAU Intraoperative Adverse Incident Classification
ED: Erectile dysfunction
EDITS: Erectile Dysfunction Inventory of Treatment Satisfaction
IIEF-5: International Index of Erectile Function 5
IQR: Interquartile Range
PP: Penile prothesis
